# A single-center retrospective observational study of modified anterolateral arthroscopic double-loop plate fixation for acute acromioclavicular joint dislocation

**DOI:** 10.1097/MD.0000000000050043

**Published:** 2026-07-31

**Authors:** Longxin An, Zilong Deng, Qiong Wu, Futian Zhang, Xiaoming Yang, Xuecheng Sun, Naibo Feng

**Affiliations:** a Weifang People’s Hospital, Shandong Second Medical University, Weifang, China.

**Keywords:** acromioclavicular joint dislocation, radiographic reduction, shoulder arthroscopy, subacromial coracoid approach, Weifang Double-loop plate

## Abstract

This study aimed to evaluate the clinical efficacy of double-loop plate fixation via the subacromial coracoid approach (modified anterolateral shoulder arthroscopy) for acute acromioclavicular joint dislocation of Rockwood Grade III or higher. A retrospective single-center observational study included 68 patients treated at Weifang People’s Hospital from October 2022 to April 2024. Outcomes included pre- and postoperative pain (Visual Analog Scale), functional recovery (Constant Score), range of motion, and radiographic reduction (coracoclavicular distance [CCD] and acromioclavicular distance [ACD]). Repeated-measures analysis of variance evaluated longitudinal changes; paired *t*-tests compared CCD and ACD between 2 days and 6 months postoperatively. *P* < .05 was considered significant. All 68 patients completed ≥ 6-month follow-up. Significant improvements were observed in Visual Analog Scale, Constant Score, and range of motion at 1, 3, and 6 months postoperatively (all *P* < .01), with stable CCD and ACD radiographically. The modified arthroscopic double-loop plate fixation shows short-term efficacy in acute Rockwood III–VI acromioclavicular joint dislocation, with pain relief, functional recovery, and stable radiographic reduction. Limitations include retrospective, single-center design, lack of comparator, and short-term follow-up; long-term efficacy requires prospective controlled trials.

## 1. Introduction

The acromioclavicular joint (ACJ), located at the top of the shoulder, is formed by the distal end of the clavicle and the acromion. This flat joint allows for limited sliding movement and is reinforced by several structures, including the coracoclavicular ligament, coracoacromial ligament, and acromioclavicular ligament. These ligaments play a crucial role in maintaining the stability of the ACJ and are essential for transmitting forces from the upper limb to the trunk.^[1]^ Additionally, muscle groups around the ACJ, such as the deltoid and trapezius, are vital for maintaining the dynamic stability of the shoulder.^[2]^

Acromioclavicular joint dislocation (ACJD) typically results from either direct or indirect trauma, such as falling onto an outstretched hand, collisions in traffic accidents, or high-impact sports injuries.^[3]^ Acute ACJD is generally defined as occurring within 3 weeks of the trauma,^[4]^ and this study specifically focuses on acute cases. The severity of the injury is classified according to the Rockwood classification system, which divides the injury into 6 grades, ranging from Grade I (ligamentous stretching) to Grade VI (complete displacement with significant soft tissue damage).^[5]^ This study includes only patients with Grade III or higher injuries.

Epidemiological studies suggest that acromioclavicular dislocations account for approximately 10% to 12% of all shoulder injuries.^[6]^ While mild dislocations can typically be managed with conservative treatment, severe Grade III–VI dislocations often require surgical intervention to restore normal anatomy and shoulder function.^[2,7]^ For athletes and manual laborers, acromioclavicular dislocation can cause significant pain, reduced mobility, and may impact both career and quality of life.^[3,8,9]^ Therefore, selecting the appropriate treatment method is essential for ensuring optimal and rapid recovery of shoulder function.

In recent years, advances in minimally invasive techniques have led to arthroscopic surgery becoming a widely used approach for treating severe acromioclavicular dislocations, offering advantages such as reduced trauma and quicker recovery times.^[10]^ Traditional open surgery, involving the clavicle hook plate, has been the standard treatment; however, it often requires a large incision, prolonged rehabilitation, and carries a higher risk of complications.^[11]^ Biz et al^[12]^ conducted a study of 100 patients with acute Rockwood type III ACJ dislocations, treated either with K-wires combined with tension band fixation or K-wires alone (group B), with a mean follow-up of 44.7 months. The results showed no significant differences in clinical outcomes between the 2 groups, and both were associated with implant migration complications. In contrast, arthroscopic surgery provides improved visualization and operative flexibility, along with the added advantages of reduced postoperative pain and shorter hospital stay.^[13,14]^ In the present study, we employed a modified anterolateral arthroscopic approach with double-loop plate fixation, which simulates the biomechanical function of the coracoclavicular ligaments by encircling both the coracoid process and the clavicle. This technique combines the advantages of minimal invasiveness and mechanical stability: the anterolateral approach requires only 3 small incisions of 0.5 to 1.5 cm, minimizing soft tissue damage, while the double-loop plate resists both horizontal shear forces (preventing posterior displacement of the clavicle) and vertical traction forces (maintaining reduction), thereby reducing the risk of implant loosening.

Based on these principles, we hypothesized that the modified anterolateral arthroscopic double-loop plate fixation would demonstrate superior radiographic stability (maintenance of coracoclavicular distance [CCD] and acromioclavicular distance [ACD]), improved functional recovery (Constant Score), and acceptable safety (low complication rate) compared with traditional open hook plate fixation or arthroscopic suture techniques, while also enabling faster rehabilitation. To test this hypothesis, we conducted a retrospective analysis of 68 patients with Rockwood grade III or higher acute ACJ dislocations who were treated at Weifang People’s Hospital between October 2022 and April 2024, evaluating functional recovery, pain relief, and maintenance of reduction.

## 2. Materials and methods

### 2.1. Inclusion and exclusion criteria (Fig. 1)

Inclusion criteria: Rockwood Grade III or above ACJD; age between 18 and 70 years; normal shoulder function prior to injury; unilateral acute closed acromioclavicular dislocation; follow-up duration ≥ 6 months; and modified shoulder arthroscopy as the surgical approach.

**Figure 1.**
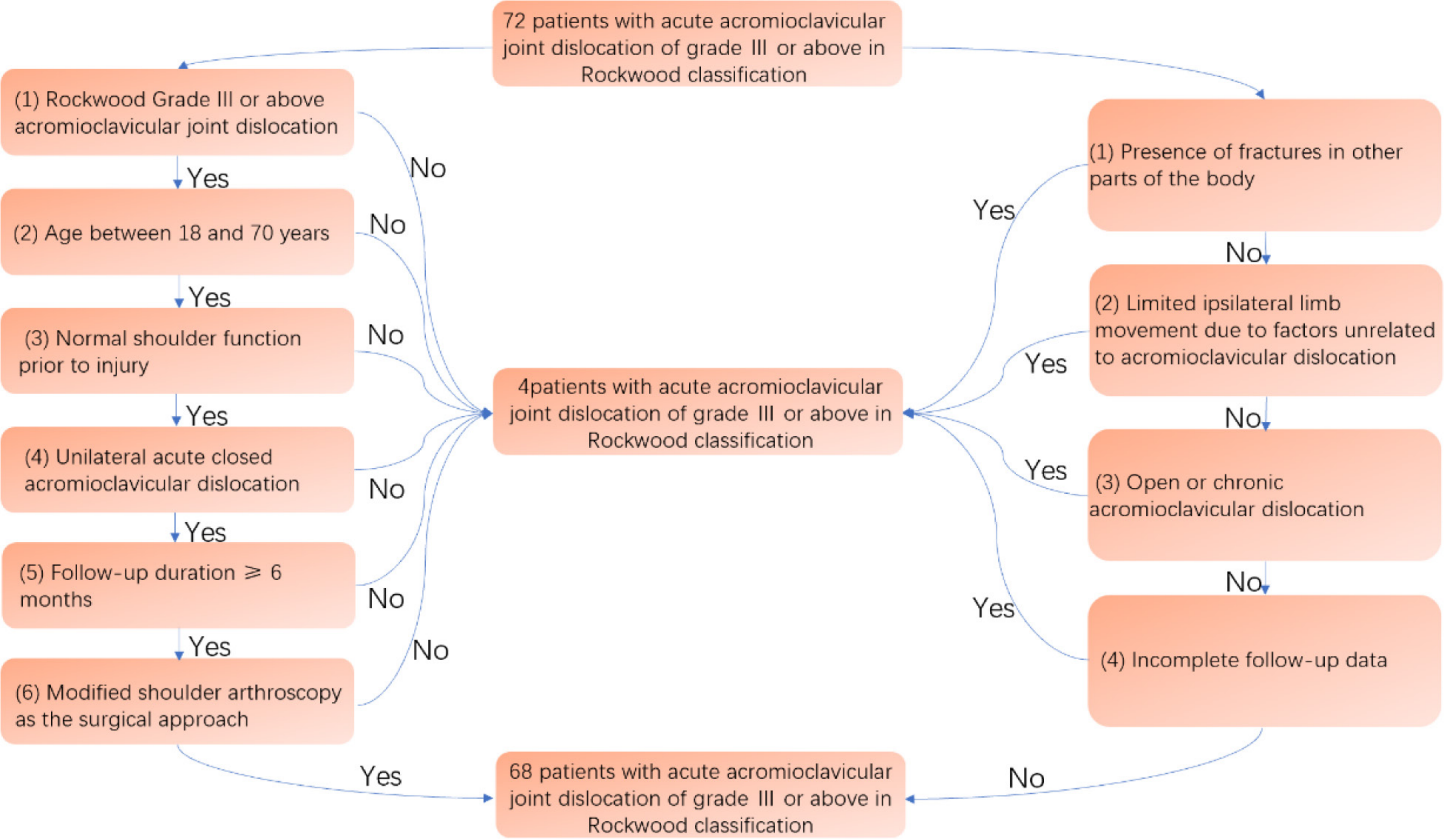
Flow diagram of patient enrollment and exclusion.

Exclusion criteria: Presence of fractures in other parts of the body; limited ipsilateral limb movement due to factors unrelated to acromioclavicular dislocation; open or chronic acromioclavicular dislocation; and incomplete follow-up data.

### 2.2. General data

A retrospective analysis was conducted on 68 patients with Rockwood grade III or higher ACJ dislocations who were admitted to Weifang People’s Hospital for traumatic injuries between October 2022 and April 2024 (Table 1). The patients’ ages ranged from adulthood to elderly, with an approximately normal distribution, and the sex ratio was balanced, minimizing potential gender-related bias. Regarding the affected limb, the predominance of injuries on the dominant side was consistent with epidemiological trends. The mechanisms of injury were almost evenly distributed between high-energy falls and traffic accidents, without overrepresentation of either type. Most cases involved moderate to severe dislocations (Rockwood grades IV–V), in accordance with the inclusion criteria, and the distribution reflects the typical clinical spectrum. This study was approved by the hospital’s Ethics Committee (approval number: KYLL20241213-1), and informed consent was obtained from all participants.

**Table 1:** Baseline characteristics of included patients.

Item	Sample size	Percentage (%)
Sex (Male/Female)	32/36	47.1/52.9
Age (yrs)	47.23 ± 12.42	–
Limb dominance (Dominant/Nondominant)	42/26	61.8/38.2
Mechanism of injury (Fall/Traffic)	35/33	51.5/48.5
Rockwood Grade (III/IV/V/VI)	9/26/29/4	13.2/38.2/42.6/5.9

### 2.3. Surgical method

**Figure 2.**
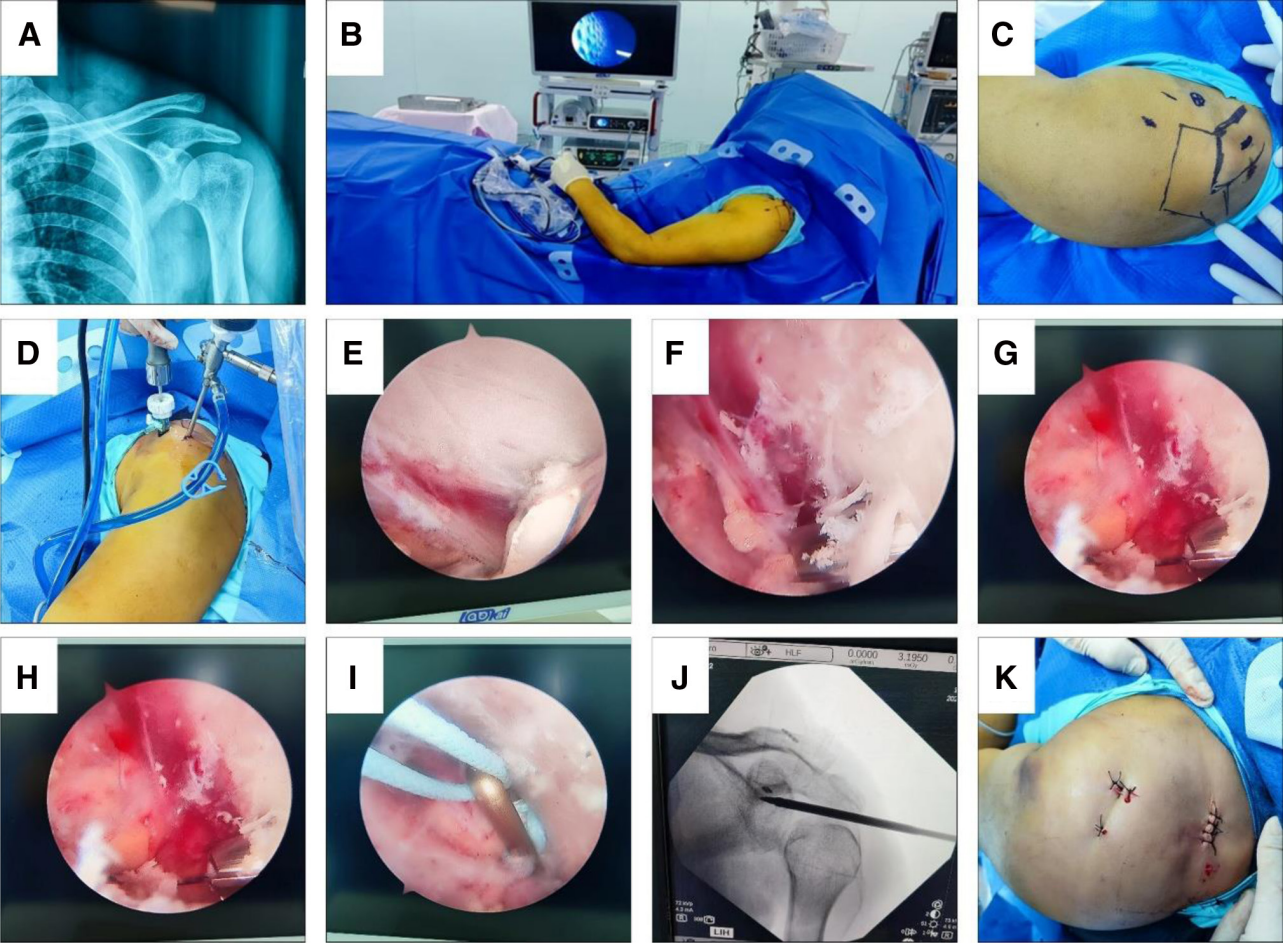
(A) X-ray of a 49-year-old male patient with left acromioclavicular joint dislocation before surgery. (B) Patient position for shoulder arthroscopy. (C) Body markings and access position. (D) Establishment of anterior and anterolateral channels. (E) Placement of cruciate ligament locator. (F) Drilling of the channel with a 4.5 mm cannulated drill. (G) Insertion of a nickel wire guide wire along the cannulated drill. (H) A traction wire with a loop plate at the tail end of the nickel wire. (I) The traction loop plate passes through the base of the coracoid process. (J) Intraoperative fluoroscopy to evaluate the reduction quality. (K) Postoperative appearance of the wound.

Preoperative X-ray films of the patient (Fig. 2A). Surgical procedures were performed under general anesthesia with endotracheal intubation, with the patient placed in the beach chair position (Fig. 2B). This position optimizes the field of vision and facilitates the surgical approach, ensuring the shoulder is positioned optimally to minimize unnecessary pressure and distortion. After routine disinfection and sterile draping of the surgical site, key anatomical landmarks, such as the coracoid process, ACJ, anterior angle of the acromion, and posterior angle of the shoulder suture, were marked (Fig. 2C).

An approximately 0.5 cm incision was made about 1.5 cm inferior and lateral to the anterior angle of the acromion to establish an observation channel for the arthroscope. This channel runs along the acromion to the base of the coracoid process. A second small incision of 0.5 cm was made slightly below the midpoint of the line connecting the anterior angle of the acromion and the coracoid process to create the anterior surgical access (Fig. 2D). The synovial tissue was cleaned using a radiofrequency device to fully expose the base of the coracoid process. Diagnostic arthroscopy was then performed to assess the condition of the ACJ and surrounding structures, allowing direct visualization of ligament tears, cartilage damage, and the type of dislocation. The scope of the injury was determined, and arthroscopic instruments were used to remove damaged tissue fragments and trim irregular bone edges, preparing the joint for subsequent repair. This step is crucial for reducing postoperative inflammatory response and promoting healing.

Next, a 1.5 cm incision was made at the distal end of the clavicle, 4 cm away from the ACJ, and a posterior cruciate ligament locator was placed (Fig. 2E). The locator’s head was placed at the center of the coracoid process base through the anterior channel. Using this locator as a guide, a 2.0 mm directional guide needle was inserted from the clavicle to the center of the coracoid process, followed by a 4.5 mm cannulated drill (Fig. 2F). A nickel wire guide was inserted along the cannulated drill (Fig. 2G), with the wire’s head pulled out through the anterior channel, and the tail was connected to the loop plate traction wire (Fig. 2H). The loop plate traction wire was drawn out through the anterior channel, and the loop plate was passed through the base of the coracoid process and flipped to form a double-loop structure, tightening the plate against the clavicle surface to reduce the ACJ (Fig. 2I). After confirming satisfactory reduction under fluoroscopy, the loop plate position was monitored under arthroscopy (Fig. 2J). The loop plate was then secured to the clavicle surface. The instruments were counted, bleeding was thoroughly controlled, the wound was sutured, and sterile dressings were applied (Fig. 2K), completing the procedure.

### 2.4. Postoperative rehabilitation plan

On the first postoperative day, passive shoulder motion exercises will be initiated under the guidance of a physical therapist. Within 2 weeks following surgery, patients are required to wear a shoulder abduction brace continuously, except during specific therapeutic sessions. After this period, patients may begin gradually incorporating active functional exercises, with a focus on closed-chain exercises – those where the hands are fixed and the arms are supported. As the range of motion (ROM) improves, patients can transition to open-chain exercises. The entire rehabilitation process typically lasts between 6 and 12 weeks.

Patients will be scheduled for follow-up evaluations at 1, 3, and 6 months post-surgery. Based on radiographic findings and assessment of shoulder joint mobility, the physician will provide individualized rehabilitation recommendations to facilitate the restoration of shoulder function.

### 2.5. Evaluation indicators

**Figure 3.**
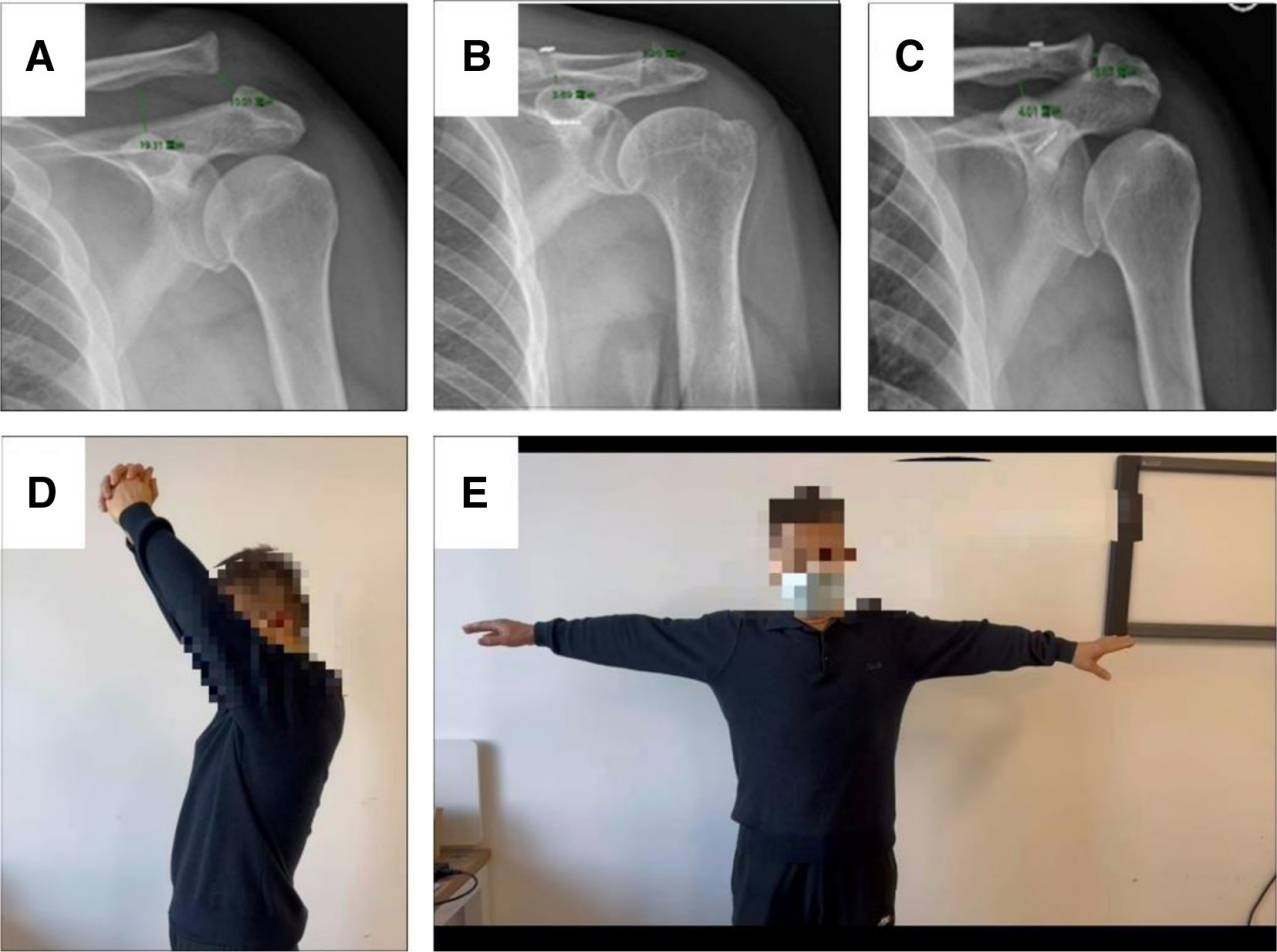
(A) ACD and CCD of the patient before surgery. (B) ACD and CCD of the patient 2 days after surgery. (C) ACD and CCD of the patient 6 months after surgery. (D) Shoulder flexion and raising of the patient after surgery. (E) Shoulder abduction and raising of the patient after surgery. ACD = acromioclavicular distance, CCD = coracoclavicular distance.

The clinical data of the 2 patient groups were recorded. The clinical outcomes were assessed using the following parameters: Visual Analog Scale (VAS) Score, Constant Score, shoulder flexion and elevation ROM (Fig. 3D), and abduction and elevation ROM (Fig. 3E). Additionally, the ACD and CCD were measured to evaluate the imaging outcomes.

### 2.6. Data collection and quality control

All outcome data were collected by an independent team separate from the surgical and clinical care personnel to minimize bias. Clinical outcomes, including the VAS, Constant-Murley Score, and shoulder ROM, were assessed by 2 attending physicians with over 5 years of experience in shoulder disorders and 1 full-time physical therapist with intermediate-level rehabilitation certification. Radiographic outcomes, including CCD and ACD, were independently measured by 2 musculoskeletal radiologists with more than 10 years of experience. Prior to the study, all assessors underwent standardized training and calibration using pilot cases or normal shoulder radiographs to ensure measurement consistency and inter-rater variability below 5%.

VAS and Constant scores were collected following standardized protocols, with patients seated in a relaxed position and marking pain on a 10-cm scale without guidance. Constant scores were assessed across 4 domains – pain, daily activities, ROM, and strength – with discrepancies resolved by a senior physician. Shoulder flexion and abduction were measured using a medical goniometer, with 2 repeated measurements averaged and a third measurement taken if the difference exceeded predefined thresholds. CCD and ACD were measured from digital radiographs using ImageJ software, with the mean of 2 independent readings recorded; a 3rd radiologist reviewed any differences exceeding 1 mm. All clinical assessors were blinded to surgical approach and Rockwood grade, while radiologists were blinded to patient information and evaluated images in randomized order to reduce observer and temporal bias.

Baseline characteristics, including comorbidities, smoking status, and pre-injury activity level, as well as all follow-up outcome measures, including the VAS, Constant Score, shoulder ROM, and radiographic measurements of the CCD and ACD, were complete for all patients. No missing data were identified for either baseline covariates or outcome variables.

### 2.7. Statistical methods

All statistical analyses were performed using Zstats software (version 1.0, https://zstats.net/). Repeated-measures analysis of variance was applied to evaluate longitudinal changes in VAS scores, Constant scores, and shoulder ROM, including flexion and abduction. Pairwise comparisons between adjacent time points for ACD and CCD were performed using paired-sample *t*-tests. A two-sided *P* value of < .05 was considered statistically significant.

## 3. Results

The follow-up duration for all 68 patients was ≥ 6 months. Follow-up results are summarized in Table 2. Repeated-measures analysis of variance was conducted to evaluate the longitudinal changes in VAS scores, Constant scores, and shoulder ROM including flexion and abduction. Mauchly test indicated that the assumption of sphericity was violated for all variables (*P* ≤ .01); therefore, the Greenhouse–Geisser correction was applied. Analysis demonstrated significant improvements in all 4 outcome measures over time (*P* < .01).

**Table 2:** Clinical data of 68 patients.

Item	VAS Score	Constant Score	Forward flexion and elevation ROM	Extensive abduction and elevation ROM
Preoperative (mean value ± SD, 95% CI)	6.65 ± 0.48, 6.53 to 6.76	35.18 ± 2.24, 34.63 to 35.72	84.35 ± 3.67, 83.46 to 85.24	74.59 ± 4.34, 73.54 to 75.64
1 mo after surgery (mean value ± SD, 95% CI)	3.65 ± 0.48, 3.53 to 3.76	74.88 ± 2.83, 74.20 to 75.57	140.53 ± 5.07, 139.30 to 141.76	114.41 ± 1.76, 113.98 to 114.84
3 mo after surgery (mean value ± SD, 95% CI)	1.47 ± 0.50, 1.35 to 1.59	124.29 ± 2.01, 123.81 to 124.78	140.53 ± 5.07, 139.30 to 141.76	168.71 ± 6.11, 167.23 to 170.19
*6 mo after surgery* (mean value ± SD, 95% CI)	0.18 ± 0.38, 0.08 to 0.27	85.41 ± 2.16, 84.89 to 85.93	168.71 ± 6.11, 167.23 to 170.19	135.29 ± 3.70, 134.40 to 136.19
Greenhouse–Geisser W, *P*	0.69, <.01	0.72, <.01	0.46, <.01	0.59, <.01
RM-ANOVA F, *P*	2963.68, <.01	6924.87, <.01	3853.56, <.01	5857.68, <.01
*6 mo after surgery*-preoperative (mean value, 95% CI)	−6.47, −6.59 to −6.34	50.23, 49.52 to 50.95	84.35, 82.60 to 86.10	61.53, 59.93 to 63.15
Partial η^2^	0.978	0.99	0.983	0.989

CI = confidence interval, MD = mean difference, ROM = range of motion, SD = standard deviation, VAS = Visual Analog Scale.

### 3.1. Imaging results

The follow-up duration for all 68 patients was ≥ 6 months. The follow-up results are summarized in Table 3. Compared to preoperative values, both the CCD and ACD, measured on X-rays before surgery (Fig. 3A) and 2 days after surgery (Fig. 3B), showed significant reduction (*P* < .01). No loss of reduction was observed in the CCD and ACD measurements between 2 days post-surgery and 6 months after surgery (Fig. 3C; CCD: *P* = .489, ACD: *P* = .06).

**Table 3:** ACD and CCD of patients before surgery, 2 days after surgery, and 6 months after surgery (n = 68).

Item	CCD (mm; mean value ± SD, 95% CI)	*T*-value	*P*-value	ACD (mm; mean value ± SD, 95% CI)	*T* -value	*P*-value
Before surgery	17.64 ± 0.37, 17.55 to 17.73			9.34 ± 0.20, 9.29 to 9.38		
		294.17	*P<.01*		164.00	*P<.01*
2 d after surgery	4.56 ± 0.20, 4.52 to 4.61			2.55 ± 0.29, 2.48 to 2.62		
		−0.70	*.489*		−2.84	*.06*
6 mo after surgery	4.57 ± 0.18, 4.53 to 4.61			2.59 ± 0.31, 2.52 to 2.67		
6 mo after surgery – before surgery (mean value ± SD, 95% CI)	−13.07 ± 0.37, −13.16 to −12.98			−6.75 ± 0.34, −6.83 to −6.67		

ACD = acromioclavicular distance, CCD = coracoclavicular distance, CI = confidence interval, SD = standard deviation.

## 4. Discussion

Various surgical techniques are available for ACJ dislocation, including ligament reconstruction, suture anchor fixation, and clavicle hook plate fixation, each with distinct advantages and limitations. Suture anchor fixation is minimally invasive but may provide limited long-term stability, whereas hook plates ensure immediate fixation but are associated with larger incisions, prolonged recovery, and higher complication rates.^[15,16]^ The choice of technique depends on injury severity, timing, surgeon experience, and patient-specific factors.

Arthroscopic surgery and clavicular hook plate fixation are commonly used approaches. Arthroscopy, as a minimally invasive technique, allows smaller incisions, reduced soft tissue trauma, lower postoperative pain, and shorter rehabilitation.^[17–21]^ Early mobilization is facilitated, potentially preventing stiffness and adhesions. Several studies report favorable functional outcomes, fewer complications, and high patient satisfaction with arthroscopic techniques.^[22,23]^ In contrast, hook plate fixation involves extensive exposure, with risks including subacromial impingement, acromial osteolysis, nerve injury, and postoperative stiffness.^[24–30]^ Although hook plates provide immediate stability, their restrictive effect may delay return to daily activities, and complications may necessitate secondary interventions. From a cost-effectiveness perspective, arthroscopic or loop plate procedures may incur higher initial costs due to specialized equipment and technical demands. However, shorter hospital stays, faster rehabilitation, and lower complication rates may reduce overall costs, whereas hook plate fixation may entail additional procedures such as plate removal, increasing total expenditure.^[31,32]^

In this study, we evaluated a modified anterolateral arthroscopic approach combined with double-loop plate fixation. The loop plate encircles the clavicle and coracoid process, mimicking the biomechanical function of the coracoclavicular ligament, resisting horizontal shear and vertical traction, maintaining anatomical reduction, and promoting soft tissue healing.^[33–35]^ Preliminary findings suggest that this technique achieves short-term radiographic stability and functional recovery, with low complication rates and relatively rapid rehabilitation. Its minimally invasive nature may allow earlier return to daily activities and improved patient-reported outcomes. Post-traumatic cartilage injury or inadequate reduction is a major contributor to long-term osteoarthritis, with an estimated incidence of 15% to 30%.^[36]^ In this cohort, the modified technique maintained anatomical reduction, minimized secondary cartilage damage, and showed no radiographic osteoarthritis signs at 6 months. Intraoperative over-reduction was avoided (CCD < 6 mm increases joint surface stress), and early standardized rehabilitation began 1 week postoperatively. High-risk patients (Rockwood V–VI or intraoperative cartilage injury) were scheduled for annual magnetic resonance imaging evaluation.

However, several limitations must be acknowledged. This is a single-center retrospective study without randomization or a comparator group; outcomes are based on pre- and postoperative self-comparisons rather than direct comparisons with other techniques. Selection bias may exist, as patients with more severe soft tissue injury or better preoperative function could have been preferentially selected, potentially slightly overestimating effectiveness. Despite strict inclusion/exclusion criteria and standardized data collection, minor postoperative events may be incompletely documented. Quality control included independent collection of clinical outcomes (VAS, Constant Score, and ROM) and blinded radiographic assessment (CCD and ACD) by experienced personnel with third-party verification; nevertheless, measurement bias cannot be entirely excluded. Subjective outcomes such as VAS may fluctuate due to patient factors, and radiographic measurements may vary slightly due to landmark identification (up to 0.3–0.5 mm).

## 5. Conclusion

In this single-center retrospective cohort, the modified technique was associated with satisfactory short-term clinical and radiographic outcomes. However, these findings should be interpreted as preliminary, and comparative prospective studies are needed to establish its role relative to existing treatments.

## Author contributions

**Conceptualization:** Xuecheng Sun, Naibo Feng.

**Data curation:** Zilong Deng, Xiaoming Yang, Xuecheng Sun, Naibo Feng.

**Formal analysis:** Xuecheng Sun, Naibo Feng.

**Methodology:** Zilong Deng, Qiong Wu.

**Supervision:** Qiong Wu.

**Validation:** Qiong Wu.

**Visualization:** Naibo Feng.

**Writing – original draft:** Longxin An, Futian Zhang, Xiaoming Yang, Naibo Feng.

**Writing – review & editing:** Longxin An, Xiaoming Yang, Naibo Feng.
